# Intra-Uterine Fractures

**Published:** 1860-05

**Authors:** E. McDonnell

**Affiliations:** N. Y. Med. Press.; 209 W. 42d Street, New York


### *Intra-Uterine Fractures.*

To the Editors of the Medical Press :

GENTLEMEN :—In the Medical Press for March 24th, 1860, you copy an article from the March No. of the New Orleans Medical News and Hospital Gazette, commenting on a case I contributed to your pages in January last. It runs thus :

" *Obstetric Phenomenon.*—Dr. E. McDonnell, of New York City, reports in the N. Y. Medical Press for January 2d, 1860, a case in which he performed version, and on examining the head of the child, the bones of which were 'unusually developed,' he discovered 'a fracture, with depression on the right parietal bone over a space of two inches in extent,'—the scalp being 'partially abraded and ecchymosed.' The Dr. says this fracture 'corresponded to the part that hitched on the brim of the pelvis, and demonstrates the force of the uterine expulsive powers.'

" Does the Dr. really believe that the uterus can push the head against the pelvic brim with such force as to fracture the bones, more especially when they are 'unusually developed?' On the contrary, has it never occurred to him that in making traction after version, he may have induced the fracture?"

I confess I was surprised on seeing the above article, for the N. O. M. News & Hospital Gazette I viewed as a valuable publication, and for its Editors—one of whom occupies a distinguished position in the profession—I entertained a high respect. I can only account for it by supposing that some mere Tyro in the profession, during the temporary absence of the Editors, occupied the Editorial chair and guided the Editorial pen, as, surely, it is only in this way we can explain such an exhibition of ignorance as that displayed in the foregoing article. I shall, however, answer the interrogatories :

To the first—I say, most decidedly I believe that the uterus

"*can push the head against the pelvic brim with such force as to fracture the bones*," especially when unusually ossified, for this condition would render them, in my opinion, more liable to the accident from their fragility, whereas, in the semi-ossified state I should suppose them, in virtue of the elasticity thence imparted, not so easily fractured.

To the second—I beg to reply, that I am totally at a loss to comprehend how traction after version could produce a fracture of the parietal bone. In this way, the bones of the extremities would be more likely to be fractured; but I do not think that this particular fracture could be so produced. Again, there was little force used in the traction of the child, for after accomplishing version, there was no further difficulty, as I left the expulsion of the child in a great measure to nature, finding her equal to the task. My instructors (the Physicians of the Rotunda Lying-in-Hospital, Dublin, in 1841,) ever inculcated the importance of exercising the greatest patience and gentleness in midwifery, and always of rather assisting than interfereing with nature; and in no case, during the last 18 years, have I more strictly obeyed those precepts than in this one. I may state, further, in support of the cause I assigned for the fracture, that the abrasion of the scalp had the appearance of being produced many hours before delivery, while version and subsequent birth of the child occupied a comparatively short time. Now, as muscular contraction is capable of fracturing the adult bone—a fact which I presume no surgeon denies—I cannot see why the powerful contractions of the uterine muscles may not fracture the infantile bone; and with all due deference and respect for the authority of the Editors of the N. O. Med. News & Hospital Gazette, *so gratuitously extended to me*, I believe this not only possible, but that it has really frequently happened, and that it is not such a "phenomenon in midwifery" as those gentlemen would have us to suppose. I shall adduce a few authorities which will have as much weight, at least with the profession, I think, as *that* which impugns my report of the case.

In the London edition of Dr. Churchill's work on the Theory and Practice of Midwifery, for 1842, we read at page 227, paragraph 462—"The child, too, may suffer considerably—if the head enter the brim and be much compressed, its life may be sacrificed, or partial pressure on any part may fracture one of the bones of the cranium, or give rise to inflammation or sloughing of the scalp."

In Malgaigne's work will be found similar testimony. In Hamilton's recent work on fractures, this language occurs:—"Fractures occurring from violence inflicted on the child by the accoucheur, or from the contraction of the neck of the womb, while the child is in transitu, are more common occurrences and do not require a separate consideration. I shall mention several in connection with the various bones in which they take place."

Did time permit, or did I not fear occupying too much space in the Medical Press, I might have referred to a number of other distinguished authors to prove my position. Doubtless the above will be considered sufficient.

In conclusion, let me remark, that if there are those who are in the habit of adopting the physical force doctrine in obstetric practice, and in their hands such an accident as here alluded to has been produced, they should not hasten recklessly to the conclusion that a similar cause occasions the accident in the practice of others, and that there is *no authority for explaining its occurrence in any other way.* E. McDONNELL.

209 W. 42d Street, New York. *N. Y. Med. Press.*

